# Laryngeal Saccular Cyst

**DOI:** 10.1155/DTE.6.91

**Published:** 2000

**Authors:** Craig Benoit, Terry Day

**Affiliations:** Department of Otolaryngology Vanderbilt University Medical Center S-2100 Medical Center North Nashville TN 37232 USA

## Abstract

We present a case report of a 66-year-old female with a laryngeal saccular cyst that was treated endoscopically. Although this is an uncommon laryngeal anomaly, when it is recognized and managed appropriately early in the course of its presentation patient complications and morbidity can be avoided. The laryngeal saccular cyst can mimic or be associated with other, more serious, laryngeal pathology, including carcinoma of the larynx. Because of the known association between carcinoma of the larynx and laryngeal saccular cysts, these lesions should be fully evaluated endoscopically and surgically excised. Direct microlaryngoscopy with the use of the operating microscope is a safe and effective method for the treatment of laryngeal saccular cysts.


*Diagnostic and Therapeutic Endoscopy*, Vol. 6, pp. 91–94
Reprints available directly from the publisher
Photocopying permitted by license only

© 2000 OPA (Overseas Publishers Association) N.V.
Published by license under
the Harwood Academic Publishers imprint,
part of The Gordon and Breach Publishing Group.
Printed in Malaysia.

### Laryngeal Saccular Cyst

CRAIG BENOIT* and TERRY DAY

*Department of Otolaryngology, Vanderbilt University Medical Center, S-2100 Medical Center North, Nashville, TN 37232, USA*

*(Received 12 May 1999; Revised 10 August 1999; In final form 7 September 1999)*

**We present a case report of a 66-year-old female with a laryngeal saccular cyst that was treated endoscopically. Although this is an uncommon laryngeal anomaly, when it is recognized and managed appropriately early in the course of its presentation patient complications and morbidity can be avoided. The laryngeal saccular cyst can mimic or be associated with other, more serious, laryngeal pathology, including carcinoma of the larynx. Because of the known association between carcinoma of the larynx and laryngeal saccular cysts, these lesions should be fully evaluated endoscopically and surgically excised. Direct microlaryngoscopy with the use of the operating microscope is a safe and effective method for the treatment of laryngeal saccular cysts.**

*Keywords:* Cyst, Laryngocele, Larynx, Microlaryngoscopy, Saccule

### INTRODUCTION

The laryngeal saccular cyst is an unusual laryngeal anomaly that can mimic or be associated with more life threatening conditions. However, when it is recognized and treated early in the course of the patient's disease, serious complications and morbidity can be avoided. We present a patient with an internal laryngeal saccular cyst, including intraoperative and histopathological photographs. It was managed safely and effectively with endoscopic excision without tracheotomy.

### CASE REPORT

A 66-year-old female presented to our clinic with a 6-month history of hoarseness and intermittent shortness of breath. She denied any symptoms of odynophagia, dysphagia, or weight loss. Her past medical history was significant for a 36-pack-year smoking history. She had no prior history of laryngeal trauma or malignancy.

On examination, she had a breathy dysphonia. There were no neck masses or palpable adenopathy. Fiberoptic laryngoscopy in the clinic revealed a

* Corresponding author. Tel.: +1(615)222-6099. Fax: +1(615)222-2384. E-mail: www.craig.benoit@mcmail.vanderbilt.edu.

pedunculated, smooth, round mass arising from the anterior aspect of the left laryngeal ventricle that prolapsed into the airway with inspiration. The true vocal folds were mobile bilaterally. Due to the size and location of the cyst, the middle one third of the left true vocal fold and the anterior commissure were not completely visualized with the flexible fiberoptic examination.

A direct microlaryngoscopy was performed under general anesthesia with the patient endotracheally incubated. The larynx was exposed via suspension laryngoscopy, and the operating microscope, with a 400-mm lens, was used to view the larynx. The mass was found to be cystic in nature and arose from a 4 mm stalk attached to the roof of the mid-anterior laryngeal ventride on the left (Fig. 1). There were no other mucosal abnormalities. The lesion was retracted medially with microlaryngeal articulating cup forceps. A mucosal incision was performed approximately 5-mm lateral to the stalk of the cyst. The base of the stalk was bluntly dissected to its origin in the roof of the laryngeal ventricle. The lesion was then sharply excised along with a 2-mm cuff of ventricular mucosa around the saccular opening. Gross inspection of the lesion revealed a soft mucosal lined sac that was approximately 5 × 5 mm containing inspissated mucous. Frozen histologic sectioning was performed to detect the presence of any malignant cells. The histology supported the diagnosis of a cyst lined with pseudostratified columnar respiratory epithelium with abundant goblet cells (Fig. 2). There was no evidence of carcinoma in the specimen.

### DISCUSSION

The laryngeal saccule is an opening in the anterior middle third of the roof of the laryngeal ventricle. It is lined by ciliated respiratory epithelium with anywhere from 50 to 100 mucous glands. The function of the saccule is the lubrication of the vibrating vocal folds [1]. Blockage of the saccule opening with continued secretion of mucous leads to a saccular cyst. The saccular opening can also become dilated, leading to retrograde filling of the false vocal cord with

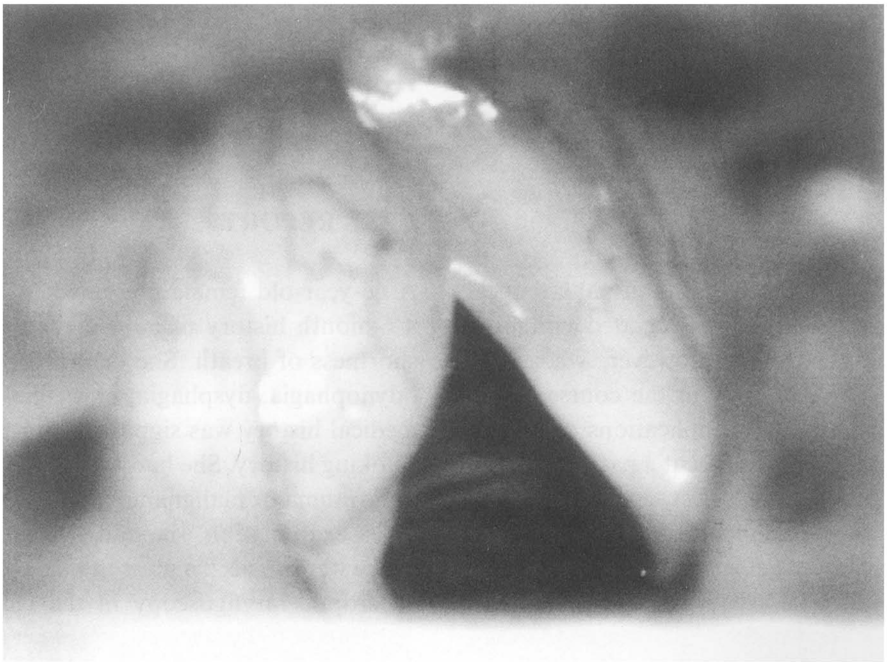

FIGURE 1 The endoscopic view of a laryngeal saccular cyst arising from the left laryngeal ventricle.

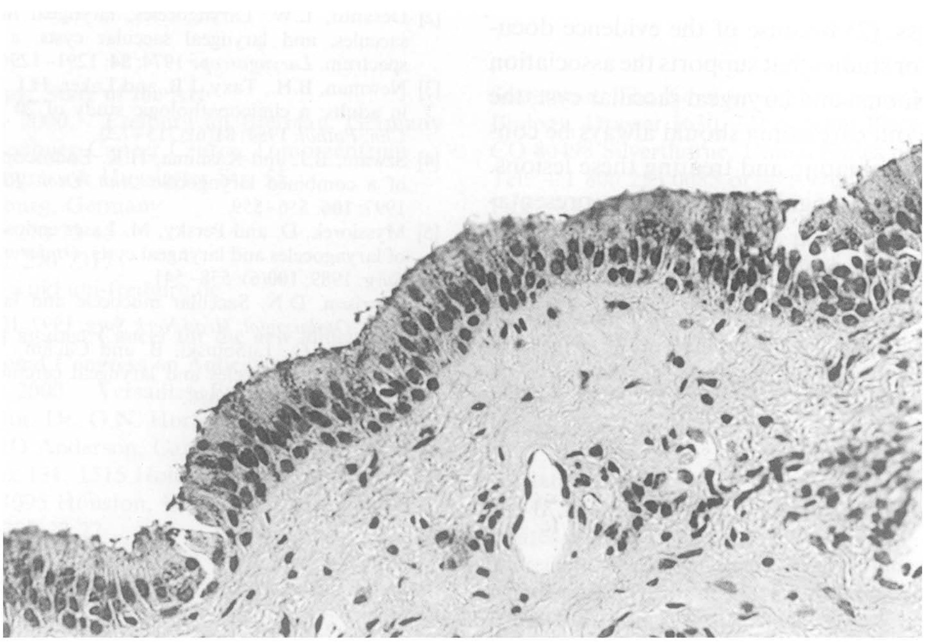

FIGURE 2 The pseudostratified columnar respiratory epithelial lining of a laryngeal saccular cyst (original ×200).

air, termed a laryngocele. The masses created by these pathologic entities can remain confined to the larynx or spread through the thyrohyoid membrane into the neck.

The classification and nomenclature of saccular cysts and laryngoceles have been well defined in the literature. Saccular cysts are mucous filled dilations of the false vocal folds that are confined to the larynx. Lateral saccular cysts extend posteriorly and superiorly to the aryepiglottic fold. Anterior saccular cysts extend medially into the laryngeal lumen between the true and false vocal fold [2].

Laryngoceles are air-filled sacs that communicate with the larynx via a dilated saccule. They are divided into three types based upon the extent of extension from the laryngeal saccule. The internal laryngocele is confined to the larynx, typically entirely within the false vocal fold. The external laryngocele is a dilation of the sac that extends through the thyrohyoid membrane at the site of the superior laryngeal neuromuscular bundle and into the neck. The air-filled mass communicates with the larynx via the dilated saccule, however, the tract through the false vocal fold is not dilated. The combined laryngocele has both an external and an internal component. In addition to containing air, laryngoceles can also contain mucous that may become secondarily infected creating a laryngopyocele.

The laryngeal saccular cyst probably represents 25% of all laryngeal cysts, with submucosal cysts of the true vocal folds being the most common [3]. They are easily managed endoscopically when properly diagnosed early in the course of the disease [4]. Several authors have advocated the use of the $CO_2$ laser [5]. Saccular cysts may be congenital or acquired in the adult by trauma, neoplasm, or inflammation. The incidence of carcinoma associated with saccular cysts and laryngoceles is well documented and ranges from 5% to 30%. The pathophysiologic process is thought to be the obstruction of the saccule by carcinoma, leading to dilation by air or inspisated secretions [6,7].

In conclusion, we present this case to illustrate several important aspects of laryngeal saccular cysts. (1) Laryngeal saccular cysts can mimic other types of more common laryngeal anomalies, and should be included in the differential of any laryngeal mass. (2) Because of the evidence documented in prior studies that supports the association between carcinoma and laryngeal saccular cyst, the intent to rule out carcinoma should always be considered when evaluating and treating these lesions. (3) Finally, when recognized early in the presentation, laryngeal saccular cysts can effectively and safely be treated endoscopically.

### *References*

[1] Holinger, L.D. *et al.* Laryngoceles and saccular cysts. *Ann. Otol. Rhino. Laryngol.* 1978; **87**: 675–685.

[2] Desanto, L.W. Laryngoceles, laryngeal mucoceles, large saccules, and laryngeal saccular cysts: a developmental spectrum. *Laryngoscope* 1974; **84**: 1291–1296.

[3] Newman, B.H., Taxy, J.B. and Laker, H.I. Laryngeal cysts in adults: a clinicopathologic study of 20 cases. *Amer. J. Clin. Pathol.* 1984; **81**(6): 715–720.

[4] Szwarc, B.J. and Kashima, H.K. Endoscopic management of a combined laryngocele. *Ann. Otol. Rhinol. Laryngol.* 1997; **106**: 556–559.

[5] Myssiorek, D. and Persky, M. Laser endoscopic treatment of laryngoceles and laryngeal cysts. *Otolaryngol. Head Neck Surg.* 1989; **100**(6): 538–541.

[6] Harrison, D.N. Saccular mucocele and laryngeal cancer. *Arch. Otolaryngol. Head Neck Surg.* 1997; **103**: 232–234.

[7] Micheau, C., Luboinski, B. and Cachin, Y. Relationship between laryngoceles and laryngeal carcinomas. *Laryngoscope* 1978; **88**: 680–688.

